# CGH-Profiler: Data mining based on genomic aberration profiles

**DOI:** 10.1186/1471-2105-6-188

**Published:** 2005-07-25

**Authors:** Falk Schubert, Bernhard Tausch, Stefan Joos, Roland Eils

**Affiliations:** 1 Theoretical Bioinformatics, Deutsches Krebsforschungszentrum, Im Neuenheimer Feld 280, 69120 Heidelberg, Germany; 2 Molecular Genetics, Deutsches Krebsforschungszentrum, Im Neuenheimer Feld 280, 69120 Heidelberg, Germany; 3 Current address: Research Group Information Systems and Semantic Web, Institute for Computer Science, University of Koblenz-Landau, Universitätsstraße 1 56070 Koblenz, Germany

## Abstract

**Background:**

*CGH-Profiler *is a program that supports the analysis of genomic aberrations measured by Comparative Genomic Hybridisation (CGH). Comparative genomic hybridisation (CGH) is a well-established, molecular cytogenetic method that allows the detection of chromosomal imbalances in entire genomes. This technique is widely used in routine molecular diagnostics. Typically, chromosomal imbalances are described in a complex syntax based on the International Standard for Cytogenetic Nomenclature (ISCN). This semantic description of chromosomal imbalances hinders a large-scale statistical analysis across different experiments, e.g. for finding aberration patterns associated with a particular disease type or state.

**Results:**

*CGH-Profiler *circumvents the semantic ISCN description by importing data from different CGH system vendors and by directly transferring the data into a table format that is readily accessible for subsequent statistical analysis. *CGH-profiler *comes with different consistency checks, calculates various statistics and automatically assigns a median copy number ratio to each chromosomal band.

Import of CGH profiles from different CGH system vendors is already supported; its extension to other systems can be readily achieved through Perl scripts.

CGH profiler can also be used to analyse comparative expressed sequence hybridisation (CESH) data. CESH reveals gene expression patterns according to chromosomal locations in a similar manner as CGH detects chromosomal imbalances.

**Conclusion:**

*CGH-Profiler *is a useful tool for processing of CGH and CESH data.

## Background

CGH (comparative genomic hybridisation) is a molecular cytogenetic method to detect chromosomal imbalances [1,2]. This technology has been widely used to study genomic imbalances with prognostic and therapeutic relevance in a variety of different diseases including cancer and mental disorders (e.g., [3,4]).

For CGH, test DNA (e.g. from tumours) and reference (normal) DNA are labelled with different fluorochromes and co-hybridised onto metaphase chromosomes from normal cells. Test and reference DNA compete for binding sites, with binding probabilities depending on the abundance of the respective DNA. When hybridising the two differently labelled DNA to a normal metaphase spread, imbalances can be detected as colour changes of the chromosomes. Quantitative measurements of the colour ratio profiles along each chromosome yields the DNA copy number differences between sample and reference DNA. Digital image processing and analysis of profiles is usually performed within commercially available CGH analysis software.

Traditionally, CGH profiles have been classified according to the International System for Human Cytogenetic Nomenclature (ISCN) [5]. ISCN is a formal language for describing DNA copy number changes, amongst others. It covers low level gains (rev ish enh), high level gain (rev ish ampl) and losses (rev ish dim). A loss of the chromosomal band 4p16 is e.g. specified as "rev ish dim(4p16)". The transformation of CGH profiles into the ISCN nomenclature is a tedious process that requires a trained molecular cytogeneticist.

Here, we describe our programme *CGH-profiler*, which circumvents the ISCN nomenclature by automatically assigning a median copy number ratio to each chromosomal band thus allowing for an automatic detection of losses, gains and high-level gains.

The program can also be used to analyse data from the more recently introduced technique called comparative expressed sequence hybridisation (CESH) [6-9]. CESH reveals gene expression patterns according to chromosomal locations in a similar manner as CGH detects copy number changes. In brief, reverse transcribed test and reference RNA are differentially labelled and co-hybridised to normal metaphase chromosomes. The resolution of CESH is low compared to microarray gene expression arrays but no prior sequence information of genes or cloning is required. Furthermore, CESH can be performed by using existing CGH / fluorescence in situ hybridisation expertise, equipment and software. Thus it appears that the CESH data format is appropriate for *CGH-profiler*.

## Implementation

The program includes the following processing steps:

• Conversion of the CGH profile values to a meta format independent of the used CGH system

• Profile cleansing, consistency check of the chromosomal length

• Interpolation to a given length (adjustable, e.g., 128 points per chromosome) by using cubic (Akima) splines

• Calculation of the median copy number change from all metaphases of a given case

• User defined exclusion of certain regions (centromeres, telomeres, tumour specific bands)

• Assignment of median copy numbers to chromosomal bands

### Profile transformation to a meta format

The CGH profile values have to be exported from a commercial CGH system. Irrespective of the CGH system, the exported profile values are then transformed to a meta format using a Perl script. A resulting meta format file includes all metaphase profiles for all chromosomes of all cases. Each metaphase profile can have a different length.

The parsing and transformation of CGH profile values from two popular CGH systems, namely CytoVision (Applied imaging, [10]) and Isis CGH (Metasystems, [11]), are supported. Profiles from other CGH system vendors may be integrated using adapted Perl scripts.

### Consistency check

A consistency check of all metaphases is performed to exclude wrongly assigned metaphases. We exclude metaphases from further analysis if the difference between its length and the mean length of the respective type of chromosome is larger than a user defined threshold (e.g. 15%). The consistency check may be switched off (by assigning 100%).

### Interpolation

The remaining metaphases are interpolated to a given length. This is a prerequisite for a consistent merge of all measurements. We used cubic Akima and Fritsch/Carlson splines (polynomials of degree 2) for this interpolation implemented in the matpack library [12]. The number of interpolation points can be defined by the user, the predefined value is 128. The predefined value of 128 is especially useful for applying a wavelet transform.

### Merging

From all metaphases of a given case and chromosome we calculate the median or mean copy number at each interpolation point. The choice of median or mean is optional to the user.

### Exclusion of bands

The CGH measurements of some chromosomal regions (e.g. those containing a large number of highly repetitive sequences) are not reliable [13], especially after PCR amplification of the probes. The measurements of certain regions should therefore not be used for an automatic analysis. We excluded all centromeres, some telomeric regions, chromosome 19 and the sex chromosomes. However, the user can specify all critical regions in a configuration file. The ratios of all excluded regions can be marked as NA or balanced.

### Assignment of median copy numbers to chromosomal bands

The mean or median profile of each case and chromosome can be mapped to an ISCN-400-ideogram without subbands [5] so that a single mean value is assigned to each chromosomal band. According to the definition of the ideogram the profile values are combined to a mean value for each chromosomal band. The predefined mapping file is based on the ISCN-400-ideogramm and a resolution of 128 interpolation points. E.g., band 1p36 is located from 1/128 to 13/128 on an ideogram. The mean value of this band is therefore the mean of the profile values 1,..,13. This data representation is the starting point for a further analysis. Using threshold values the median copy numbers can be readily translated into semantic expressions, namely losses (threshold <0.75), gains (threshold >1.25), high level gains (threshold >2) and balanced.

## Results and discussion

CGH is a well-established and still widely used method. More than 150 new CGH studies were published in 2004 and referenced in Medline.

Here, we presented the program *CGH-profiler *that allows the input of profile values from different commercial CGH systems and transforms these values into a format that can be readily used for a quantitative analysis. Notably, our program circumvents the widely used semantic notation in the ISCN standard. Thus, it provides a basis for a more accurate and reproducible interpretation of data from large-scale genomic aberration screens.

We compared losses and gains automatically detected by CGH-profiler with those described by conventional CGH analysis (encoded in ISCN) for two data sets (data not shown) and found a high degree of accordance. Notably, conventional CGH evaluation often characterises large regions as gain or loss whereas the ratio value as determined by the programme *CGH-profiler *is only altered in part of the entire region.

Data mining of CGH profiles requires a matrix representation of CGH profiles. An alternative to our approach is an ISCN-to-matrix parser [14]. This is useful for large repositories of CGH studies (e.g., Progenetix [15], providing more than 10818 cases from 383 publications, SKY [16], or Charite CGH database [17]). However, a direct transformation of profile values to a matrix representation is more efficient.

The program *CGH-profiler *has only been used for CGH-analysis in humans so far. An extension to CGH profiles in other species can be easily achieved by adopting the mapping file used for band assignment. CGH profiler can also be used to analyse comparative expressed sequence hybridisation (CESH) data.

## Conclusion

*CGH-Profiler *assigns to each chromosomal band a median copy number ratio by importing and processing data from different CGH system vendors. Data analysis of these continuous variables is much more efficient compared to the semantic descriptions defined by ISCN. *CGH-Profiler *supports therefore the data mining process of CGH and CESH data and enhances the use of mathematical functions (e.g., wavelets) on CGH and CESH profiles.

## Abbreviations

CGH-Comparative genomic hybridisation

CESH comparative expressed sequence hybridisation

## Authors' contributions

SJ and FS developed the method. FS and RE drafted the paper. BT and FS implemented the method. RE supervised the study. All authors prepared, read and approved the final manuscript.

## Availability and requirements

The program *CGH-profiler *is written in C++ while the preceding data transformation is done in Perl. It is available under the GPL-license for non-commercial purposes.

-Project name: CGH-profiler

-Project home page: 

-Operating systems: Unix

-Programming language: C++, Perl

-License: GNU GPL

-Restrictions to use by non-academics: on request

## References

[B1] Kallioniemi A, Kallioniemi OP, Sudar D, Rutovitz D, Gray JW, Waldman F, Pinkel D (1992). Comparative genomic hybridization for molecular cytogenetic analysis of solid tumors. Science.

[B2] du Manoir S, Speicher MR, Joos S, Schrock E, Popp S, Dohner H, Kovacs G, Robert-Nicoud M, Lichter P, Cremer T (1993). Detection of complete and partial chromosome gains and losses by comparative genomic in situ hybridization. Hum Genet.

[B3] Monni O, Hyman E, Mousses S, Barlund M, Kallioniemi A, Kallioniemi OP (2001). From chromosomal alterations to target genes for therapy: integrating cytogenetic and functional genomic views of the breast cancer genome. Semin Cancer Biol.

[B4] Lichter P, Joos S, Bentz M, Lampel S (2000). Comparative genomic hybridization: uses and limitations. Semin Hematol.

[B5] Mitelman F, Ed (1995). ISCN 1995: An International System for Human Cytogenetic Nomenclature (1995).

[B6] Lu YJ, Williamson D, Clark J, Wang R, Tiffin N, Skelton L, Gordon T, Williams R, Allan B, Jackman A (2001). Comparative expressed sequence hybridization to chromosomes for tumor classification and identification of genomic regions of differential gene expression. Proc Natl Acad Sci U S A.

[B7] Lu YJ, Williamson D, Wang R, Summersgill B, Rodriguez S, Rogers S, Pritchard-Jones K, Campbell C, Shipley J (2003). Expression profiling targeting chromosomes for tumor classification and prediction of clinical behavior. Genes Chromosomes Cancer.

[B8] Gruszka-Westwood AM, Horsley SW, Martinez-Ramirez A, Harrison CJ, Kempski H, Moorman AV, Ross FM, Griffiths M, Greaves MF, Kearney L (2004). Comparative expressed sequence hybridization studies of high-hyperdiploid childhood acute lymphoblastic leukemia. Genes Chromosomes Cancer.

[B9] Vanhentenrijk V, De Wolf-Peeters C, Wlodarska I (2004). Comparative expressed sequence hybridization studies of hairy cell leukemia show uniform expression profile and imprint of spleen signature. Blood.

[B10] Applied imaging. http://www.appliedimagingcorp.com.

[B11] Metasystems. http://www.metasystems.de.

[B12] Matpack library. http://www.matpack.de.

[B13] Kallioniemi OP, Kallioniemi A, Piper J, Isola J, Waldman FM, Gray JW, Pinkel D (1994). Optimizing comparative genomic hybridization for analysis of DNA sequence copy number changes in solid tumors. Genes Chromosomes Cancer.

[B14] Baudis M, Cleary ML (2001). Progenetix.net: an online repository for molecular cytogenetic aberration data. Bioinformatics.

[B15] Progenetix CGH database. http://www.progenetix.net.

[B16] SKY CGH database. http://www.ncbi.nlm.nih.gov/sky/.

[B17] Charite CGH database. http://amba.charite.de/~ksch/cghdatabase/index.htm.

